# Retraction: Mammalian STE20-like kinase 2 promotes lipopolysaccharides-mediated cardiomyocyte inflammation and apoptosis by enhancing mitochondrial fission

**DOI:** 10.3389/fphys.2022.1006549

**Published:** 2022-08-04

**Authors:** Frontiers Editorial Office

The Publisher retracts the cited article.

Following publication, the publisher uncovered evidence that false identities were used in the peer-review process. The assignment of a fake reviewer was confirmed by an investigation, conducted in accordance with Frontiers’ policies and the Committee on Publication Ethics (COPE) guidelines.

The investigation also uncovered concerns about the presentation and validity of the data in the article that normally would have led to a rejection. When contacted, the authors failed to provide a data set that adequately supports the reported conclusions.

The authors do not agree to this retraction.

This retraction was approved by the Chief Editors of Frontiers in Physiology and the Chief Executive Editor of Frontiers.

